# A prolonged, community-wide cholera outbreak associated with drinking water contaminated by sewage in Kasese District, western Uganda

**DOI:** 10.1186/s12889-017-4589-9

**Published:** 2017-07-18

**Authors:** Benon Kwesiga, Gerald Pande, Alex Riolexus Ario, Nazarius Mbona Tumwesigye, Joseph K. B. Matovu, Bao-Ping Zhu

**Affiliations:** 1 Uganda Public Health Fellowship Program – Field Epidemiology Track, Kampala, Uganda; 2 Makerere University School of Public Health, P.O. Box 7072, Kampala, Uganda; 3 United States Centres for Disease Control and Prevention, Atlanta, USA

**Keywords:** Cholera, Outbreak, Case-control studies, Uganda

## Abstract

**Background:**

In May 2015, a cholera outbreak that had lasted 3 months and infected over 100 people was reported in Kasese District, Uganda, where multiple cholera outbreaks had occurred previously. We conducted an investigation to identify the mode of transmission to guide control measures.

**Methods:**

We defined a suspected case as onset of acute watery diarrhoea from 1 February 2015 onwards in a Kasese resident. A confirmed case was a suspected case with *Vibrio cholerae* O1 El Tor, serotype *Inaba* cultured from a stool sample. We reviewed medical records to find cases. We conducted a case-control study to compare exposures among confirmed case-persons and asymptomatic controls, matched by village and age-group. We conducted environmental assessments. We tested water samples from the most affected area for total coliforms using the Most Probable Number (MPN) method.

**Results:**

We identified 183 suspected cases including 61 confirmed cases of *Vibrio cholerae* 01; serotype *Inaba*, with onset between February and July 2015. 2 case-persons died of cholera. The outbreak occurred in 80 villages and affected all age groups; the highest attack rate occurred in the 5–14 year age group (4.1/10,000). The outbreak started in Bwera Sub-County bordering the Democratic Republic of Congo and spread eastward through sustained community transmission. The first case-persons were involved in cross-border trading. The case-control study, which involved 49 confirmed cases and 201 controls, showed that 94% (46/49) of case-persons compared with 79% (160/201) of control-persons drank water without boiling or treatment (OR_M-H_=4.8, 95% CI: 1.3–18). Water collected from the two main sources, i.e., public pipes (consumed by 39% of case-persons and 38% of control-persons) or streams (consumed by 29% of case-persons and 24% control-persons) had high coliform counts, a marker of faecal contamination. Environmental assessment revealed evidence of open defecation along the streams. No food items were significantly associated with illness.

**Conclusions:**

This prolonged, community-wide cholera outbreak was associated with drinking water contaminated by faecal matter and cross-border trading. We recommended rigorous disposal of patients’ faeces, chlorination of piped water, and boiling or treatment of drinking water. The outbreak stopped 6 weeks after these recommendations were implemented.

## Background

Cholera is an acute enteric infection characterized by sudden onset of profuse, painless watery diarrhoea and vomiting. If left untreated, the disease can quickly lead to severe dehydration and death [1]. With proper and timely management, the case-fatality rate can be lower than 1% [1]. Cholera is caused by toxigenic *Vibrio cholerae* serogroup O1 and O139. Serogroup O1 occurs as 2 biotypes: Classical and El Tor. Each biotype can occur as 3 serotypes: *Inaba, Ogawa and Hikojima*. The gold standard for cholera diagnosis is stool culture. However in resource-limited settings, the Crystal VC® dipstick rapid test can be used to alert public health officials of a possible cholera outbreak [2]. However, this dipstick rapid test has suboptimal sensitivity and specificity; therefore faecal specimens tested positive for *V. cholerae* using this Rapid Diagnostic Test (RDT) should be confirmed using the traditional culture-based methods [2]*.*

Globally, an estimated 1.4 to 4.3 million cholera cases and 28,000 to 142,000 cholera-related deaths occur every year [1]. The disease incidence declines as communities develop and access to clean drinking water and food improves [1]. Currently, cholera outbreaks mostly occur in developing countries, especially Sub-Saharan Africa. In 2008, the World Health Organization (WHO) classified 51 countries as “endemic” for cholera because they had reported cholera cases in at least 3 of 5 most recent years [3]. Most of these 51 endemic countries are in Sub-Saharan Africa, where cholera outbreaks continue to occur repeatedly [3]. In Uganda, which is one of the 51 endemic countries, cholera outbreaks occur mainly along the western border with Democratic Republic of Congo (DRC), in the Karamoja region to the north and in Kampala City slums [4]. Uganda has a high incidence and prevalence of diarrheal diseases, especially in slums and rural areas [5]. In 2011 the prevalence of diarrhoea among children under 5 years of age in Uganda was approximately 30% [6]. Lack of safe drinking water and poor sanitation contributed to the high diarrheal disease burden. In Uganda, 8% of people drink surface water while 7% practice open defecation [7]. In one area, improved Water, Sanitation and Hygiene (WASH) coverage significantly reduced the number of diarrheal disease outbreaks [8].

The most common diarrheal diseases among Ugandan children under 5 years of age are typhoid, dysentery, and rotavirus infection [9]. The WASH coverage in Kasese District is similar to the rest of rural Uganda. According to the district’s annual health reports, the safe water coverage and latrine coverage are both at approximately 60% [10]. This low WASH coverage leads many families to use streams as water sources as well as defecation points, increasing the risk of water-borne disease outbreaks.

Kasese District has a hilly terrain; hence many areas are supplied by gravitational water flow schemes (GWFS). To ensure the water in GWFS is safe for human consumption, one must chlorinate the water and check its quality frequently; yet these measures are not implemented regularly. In addition, several vibrant fish markets operate in the western part of the district near the border with DRC. Poor hygiene and sanitation conditions in these markets and busy cross-border trading activities usually create an ideal environment for disease spread [11]. Consequently, the district is prone to waterborne diseases, including cholera. Since 2000, the district has had 2 other cholera outbreaks prior to the current outbreak [4, 5].

Surveillance of diarrhoea diseases in Uganda follows the WHO’s Integrated Disease Surveillance and Response (IDSR) guidelines [12, 13]. Community health workers routinely report cases of acute diarrhoea to the nearest health facility, which in turn reports the data to the Uganda Ministry of Health (MoH) through the electronic Health Management Information System. The data are analysed weekly for outbreak detection [9]. When an unusually high number of cases of acute watery diarrhoea are reported from an area, investigation and laboratory confirmation are conducted. For cholera, if 1 case is culture-confirmed, an outbreak is declared, and the affected district should initiate an investigation and response with the support of MoH [12].

On 14 March 2015, the Kasese District Health Officer notified MoH of a cholera outbreak. The first reported case-person, a 12-year-old boy, had symptom onset on 14 March 2015. On 15 March 2015, he tested positive for *Vibrio Cholerae* using the Crystal VC® dipstick rapid test. This case was later culture-confirmed to be *Vibrio Cholerae* O1, biotype El Tor, serotype *Inaba.* Prior to his symptom onset, this case-person reportedly had crossed the border to DRC, where anecdotally a cholera outbreak had occurred recently. Despite efforts by the district’s local team, the outbreak continued to spread from village to village. On 15 May 2015, MoH assembled a team to support the district in investigating and controlling the outbreak. This report summarizes findings from the epidemiologic investigation, which aimed at identifying the mode of transmission so as to guide control measures.

## Methods

### Descriptive epidemiology

Kasese District is located in the western part of Uganda. It has 20 rural sub-counties and 4 towns with 115 parishes/wards and 656 villages. According to the 2014 National Census, Kasese District has a population of 702,029 people, including 338,796 males (48%) and 363,233 females (52%) [10]. The majority (75%) of the population lives in rural areas. Bwera Sub-County, where the first batch of cases reportedly occurred, is located about 5 km from the border with DRC, and has multiple markets with bustling cross-border trading.

After reviewing the first cases and identifying the commonest signs and symptoms, we constructed a two-tiered case definition as follows. A suspected case was onset of acute watery diarrhoea in a Kasese District resident from 20 February 2015 onwards. A confirmed case was a suspected case with *V. cholerae* identified from the patient’s stool specimen by culture.

To find cases systematically, we reviewed patient records kept at Bwera Hospital from 1 March 2015 onward to identify suspected cases. The patient records contained basic information for each patient, i.e., name, age, sex, residence, date of admission, date of hospitalization, and symptoms. We also reviewed data in the Health Management Information System, an electronic health data reporting system managed by MoH, on cholera cases reported in the area [9]. We then interviewed suspected case-patients to collect information on their food and water consumption history using a standardized questionnaire. We also worked with other health facilities and members of Village Health Team to actively identify cases using the standard case definition. The local public health authorities set up 3 cholera treatment centres in the most affected areas, where all suspected cases were managed with oral and intravenous fluid rehydration and antibiotics. We line-listed all patients at the cholera treatment centres. We performed descriptive analysis of the data in the line-list on patients’ clinical presentations, as well as distribution of the cases by patients’ age, sex, place of residence, education level, and used epidemic curves to describe case-patients’ dates of onset.

### Case-control study

Based on the findings from the descriptive analysis, we developed a hypothesis about the most likely mode of transmission. To test the hypothesis, we conducted a case-control study in the most affected areas, i.e., Bwera and Kitswamba sub-counties.

We recruited confirmed case-persons in the case-control study. If a household had 2 or more eligible case-persons, we only interviewed one. For each case-person, we selected 4 control persons. A control-person was an individual who never had diarrhoea from February 2015 to the time of the investigation, resident in the same village as the case-person and within the same 5-year age group as the case-person. To select control-persons randomly, we obtained a list of the households in each village with eligible cases, and wrote the names of the household heads on paper lots. We randomly drew out four times as many paper lots as the number of cases in the village, and attempted to identify an eligible control-person from each of the selected households. If not enough controls were found, we repeated the process until we had found enough controls.

We administered a questionnaire to the eligible case- and control-persons to obtain information on their food and water exposures. Data were collected on the respondents’ water source, whether the respondents treated (using chlorine tablets) or boiled their drinking water, what kind of food they usually ate, whether they usually ate hot or cold food, as well as demographic variables (i.e., age, sex, place of residence, place of work, occupation). We used the 2014 Uganda Population and Housing Census data on populations up to parish level to calculate attack rates. We stratified the data by age-group and village to obtain Mantel-Haenszel odds ratios (OR_M-H_). We trained 6 health workers from Bwera Hospital on how to identify case- and control-persons and how to administer the questionnaire.

### Laboratory investigations

We collected stool samples from suspected case-persons and placed the samples in plastic containers. Using swabs, stool samples were placed in Cary–Blair medium, and transported to the Bwera Hospital Laboratory in Kasese. Stool samples were first tested using a cholera RDT kit (Crystal VC™, EIKEN, Japan) and later by stool culture. The RDT was performed according to the instructions that came with the package [2]. Approximately 4–6 drops of liquid stool was transferred to a test-tube using a pipette that comes with the dipstick package. The dipstick was then inserted into the liquid stool, and the results were read after approximately 15 min. The dipstick had a positive test band and a control band. A test was judged as positive if both the test and control bands appeared. If only the control band appeared, it was judged as negative; if the control band did not appear, the test was judged as invalid [2].

To perform microbial analysis of the stool, the swabs were first cultured on Thiosulphate-Citrate-Bile-Salts Sucrose (TCBS™; EIKEN Japan) agar inoculated in alkaline peptone water at 37 degrees Celsius for 18–24 h. Upon identification, the *V. cholerae* isolates were further evaluated to ascertain the serogroup and serotype by agglutination with Polyvalent O1 and monospecific Ogawa and Inaba antisera [14, 15]. The RDT provided rapid diagnosis and was crucial in the initial phase of the investigation. The culture method provided confirmative diagnosis. Both were performed by trained laboratory personnel.

### Environmental investigations

Water contaminated by sewage has been implicated in several previous cholera outbreaks [16, 17]. Hence we inspected drinking water sources in the most affected areas for possible faecal contamination. The main piped water source in Bwera, the most affected Sub-County, was a GWFS. The scheme started from the top of one of the hills, where the water was chlorinated for consumption. Driven by gravity, the water ran down a pipeline system and was distributed to public and private (household taps) users throughout Bwera Sub-County.

We systematically collected water samples along the GWFS pipeline and in households using sterile containers. Samples were collected from the starting point of the GWFS, and at 2 other points along the pipeline. We also collected samples at 2 public and 2 household water taps and from water containers in 6 randomly selected households. We used the Most Probable Number (MPN) method to determine the presence of faecal coliforms in the water samples, as indicators of sewage contamination [18, 19]. To perform the test, we pipetted 10 ml of water sample into a dilution bottle containing 90 ml of phosphate-buffered dilution water. To prepare a 1/100 dilution, we mixed the 1/10 dilution bottle well and pipetted 10 ml of its contents into a bottle containing 90 ml of dilution water. Subsequent dilutions were then made in a similar way resulting in dilutions from 1/10 to 9/10. The tubes were labelled with the sample reference number and the volume of sample (or dilution) was added to the tube. They were shaken gently and the rack was placed in a water-bath for 48 h at 37 degrees Celsius. After 18–24 h tubes that displayed colour change were regarded as presumptive positive. The number of positive tubes at each dilution was recorded. After 48 h, we prepared tubes of coliform culture medium for confirmation of faecal coliforms. Using a sterile wire loop, we transferred inoculant from each presumptive positive tube into coliform medium tubes. We labelled and incubated these tubes as before. We then noted the tubes that showed growth with the production of gas as positive. Lastly, we compared the pattern of positive results with a most probable number table to obtain the final result for each sample [20].

## Results

### Descriptive epidemiology

**Fig. 1:**
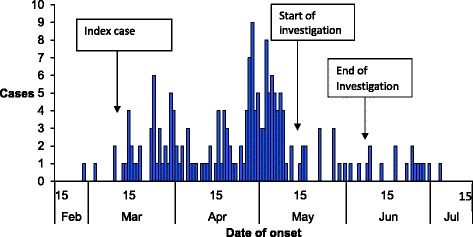
Distribution of symptom onset dates of cholera cases: Kasese District, Uganda, February to July 2015

The outbreak lasted 5 months, from February to July 2015. By 30 June 2015, 183 suspected cases had been identified in the district, including 2 deaths (Fig. 1). Cases occurred in more than 80 villages throughout the district. Most of the cases were from 2 sub-counties: Bwera and Kitswamba. The commonest symptoms were diarrhoea (100%), vomiting (46%), abdominal pain (40%) and fever (2%) (Table 1).

**Table 1:** Distribution of symptoms among 183 suspected cholera case-persons: Kasese District, Uganda, February to July 2015

Symptoms	Frequency	Percent
Acute watery diarrhoea	183	100
Abdominal pain	74	40
Vomiting	84	46
Fever	4	2.2

The median age of the case-patients was 18 years (range: 1–90 years). The age group 5–14 was the most affected (attack rate [AR]: 4.1/10,000), followed by the age groups <5 years (AR: 3.7/10,000) and 15–24 years (AR: 2.7/10,000). Males (AR: 2.6/10,000) and females (AR: 2.6/10,000) were equally affected (Table 2).

**Table 2:** Age- and sex- characteristics of 183 suspected cholera case-persons during a cholera outbreak: Kasese District, Uganda, February to July 2015

Characteristic	Number	Population	Attack Rate (10,000)
Age (years)			
< 5	28	70,203	4.0
5–14	47	112,325	4.2
15–24	37	116,537	3.2
≥ 25	71	402,965	1.8
Sex			
Male	91	338,796	2.7
Female	92	363,233	2.5
Totals	183	702,029	2.6

**Fig. 2:**
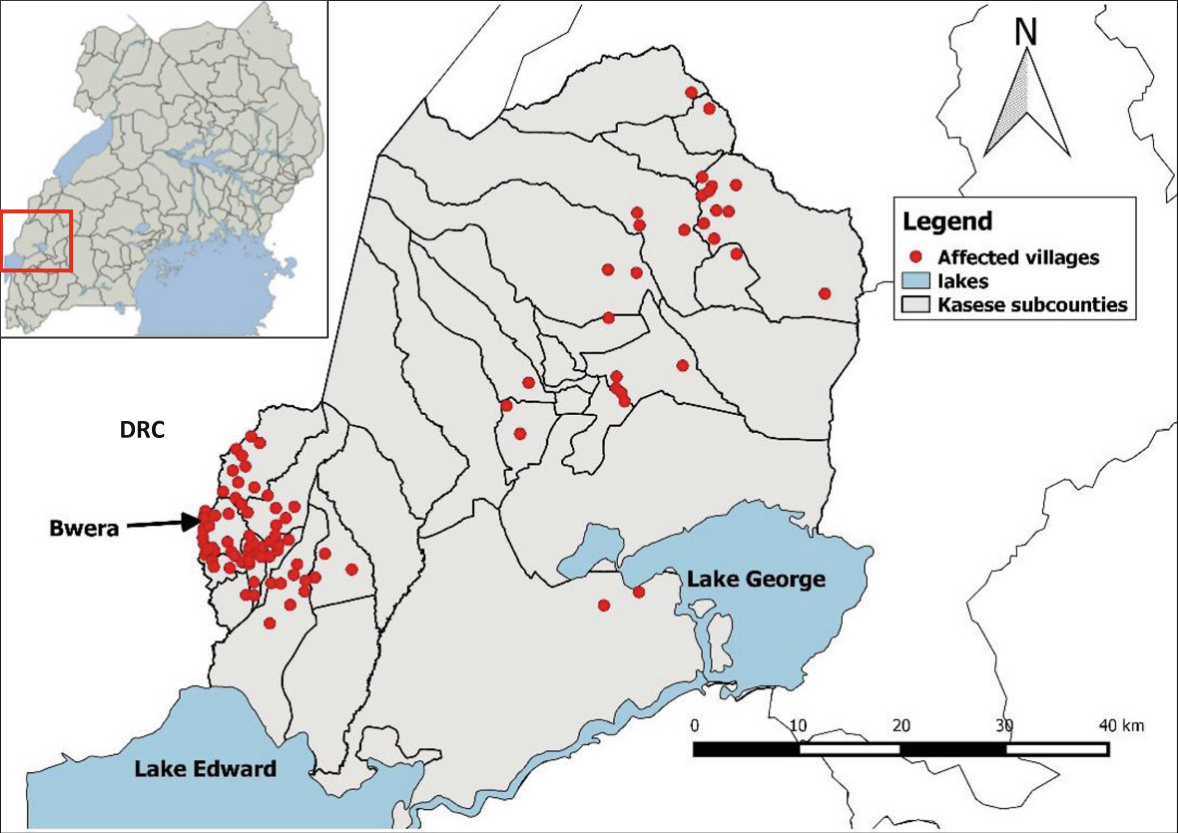
Map of villages affected by a cholera outbreak: Kasese District, Uganda, February to July 2015. Inset is the map of Uganda showing the location of Kasese, the district where the cholera outbreak occurred

The outbreak started in villages in Bwera Sub-County near the DRC border, spread eastward through Kasese municipality to Kitswamba Sub-County. In total, more than 80 villages across Kasese District were affected. The highest attack rates were found in parishes within Bwera and Kitswamba sub-counties (Fig. 2). Stratified epidemic curves revealed small clusters of cases occurring in the villages as the outbreak spread eastward.

### Case-control study findings

In the case-control investigation, 34% (17/49) of case-persons compared to 32% (64/201) of control-persons obtained their drinking water from a river or stream (OR_M-H=_1.3, 95% CI: 0.57–2.8). However, 94% (46/49) of case-persons compared with 76% (160/201) of control-persons drank water deemed unsafe, i.e., water that was neither treated nor boiled (OR_M-H_ = 4.8, 95% CI: 1.3–18). None of the other potential exposures, e.g., eating fish, and from which market were the fish usually bought, or eating cold food, were associated with cholera (Table 3).

**Table 3:** Distribution of exposure status among cases and controls during a cholera outbreak in Kasese between February and July 2015

Exposure	Num. of participants		% exposed		OR^a^_M-H_ (95% CI)
	Cases (*n* = 49)	Controls (*n* = 201)	Cases	Controls	
Drinking unboiled/untreated water	46	153	94	76	4.8 (1.3–18)
Source of drinking water: River or Stream	17	64	34	32	1.3 (0.57–2.8)
Eating fish	48	187	98	93	3.4 (0.48–24)
Market where fish was bought					
Customs market	12	70	25	35	0.39 (0.13–1.2)
Mpondwe market	15	56	30	28	1.2 (0.43–3.5)
Musyenene market	6	9	13	5	4.2 (0.69–25)
Sex (males)	23	111	47	55	0.66 (0.33–1.3)
Education (≤Primary School)	10	20	20	10	2.3 (0.97–5.6)

^a^Adjusted for age group and village, using the Mantel-Hanszel method

### Laboratory findings

In total, 78 stool samples collected from suspected case-patients tested positive for cholera by RDT, of which 61 were culture-positive for *V. cholerae* O1, biotype El Tor serotype *Inaba*. We did not test all suspected cases because of insufficient supplies of the RDT kits; also, according to WHO guidelines, in resource limited settings like Uganda, it is not necessary to test all suspected cases after the initial cases have been confirmed [1].

Water testing showed that, in samples taken from the water chlorination point of the GWFS, the total coliform count was ≤10 MPN/100 ml [18]. This level meets Uganda’s standard for safe drinking water (≤ 10MPN/100 ml). However, samples collected further along the pipeline from the public water taps in the communities had a total coliform count of 200 MPN/100 ml, while samples from household containers had a total coliform count of ≥800 MPN/100 ml.

### Environmental assessment findings

Bwera sub-County, the most affected sub-County, borders DRC. High volumes of cross-border trading occur daily. The main water supply for this area is GWFS water from River Rubiriha, which marks the border between Uganda and DRC. Many people collected their drinking water from this river. We inspected the GWFS and found that the water was insufficiently chlorinated for safe drinking. We also found points of leakage and potential contamination along the pipeline. We observed that many families were washing their clothes and kitchen utensils in the rivers and streams. Most homes did not have proper toilet or pit latrine facilities. Many people, especially young children, swam and defecated in the water.

## Discussion

The cholera outbreak we investigated was the third such outbreak in Kasese District since 2000; with all of them starting from Bwera Sub-County [4, 12]. This outbreak lasted 5 months and affected 183 people. Our epidemiologic and laboratory investigation and environmental assessment demonstrated that the outbreak was caused by drinking contaminated water, as is often the case with cholera outbreaks [21].

During this outbreak, the river water in the affected areas was highly contaminated with faecal matter; thus it was unsafe to drink directly. The insufficiently chlorinated public tap water in Bwera Sub-County was supplying most of the affected areas. Unsafe drinking water has repeatedly been implicated in cholera outbreaks in the past [16, 17]. During an investigation conducted among semi-nomadic pastoralists in Karamoja, Northern Uganda, drinking untreated water was associated with developing cholera [22]. In a rural village in India, a damaged pipeline was contaminated by sewage, causing a cholera outbreak [23]. We also demonstrated that, after adequately chlorinating the public tap water, the outbreak stopped, as in previous outbreaks [17, 24]. Water treatment has been shown to help prevent up to 90% of waterborne diseases, including cholera [25].

Cholera outbreaks had occurred multiple times in Bwera Sub-County, Kasese District, yet they had not been investigated comprehensively and epidemiologically. Our findings could shed light on why cholera outbreaks repeatedly occurred in this area. The first case-person visited DRC (where a cholera outbreak reportedly occurred) before symptom onset, suggesting that he might have contracted the disease on the DRC side. After cholera was introduced into the community, unsafe drinking water and poor hygiene likely helped to spread the disease during this and previous outbreaks. In fact, during previous outbreaks, unsafe water and cross-border activities were also cited as possible exposures, although this was not substantiated by detailed epidemiologic investigations. These risk factors persist in the area; therefore new outbreaks could again occur in the future if they are not addressed.

After the first case was confirmed, there was a delay of about a month before an outbreak investigation and comprehensive control efforts were launched. Prompt investigation and timely implementation of control measures have been shown to reduce the extent and scope of outbreaks. For example, Mwambi et al. showed how a prompt investigation and adequate response helped to contain a cholera outbreak in Zambia within a short period of time [26]. The chain of transmission was interrupted through proper disposal and disinfection of patients’ faeces, fixing the piped water system and advising people to boil or treat their drinking water. Cross-border coordination and collaboration are an important strategy for preventing the spread of infectious diseases from one country to the other. During this outbreak, even though anecdotal cholera outbreaks were occurring simultaneously on both sides of the border, little information exchange between Uganda and DRC took place. Moreover, there was no official cross-border coordination to respond to this outbreak. Improved coordination from both sides is needed in the future so that outbreaks can be detected, responded to, and contained earlier [27].

Although a cholera vaccine is currently available, its effectiveness for control of cholera in endemic and epidemic areas has been mixed. Cholera vaccination has been shown to be effective for controlling outbreaks only when it was used together with safe water provision and sanitation improvement [28, 29]. On the other hand, primary prevention strategies, such as improvement of sanitation, waste disposal, and provision of safe water, are the most effective and sustainable interventions not only against cholera [22] but also against other waterborne diseases.

### Limitations

We did not follow WHO guidelines for sampling of environmental water points for testing; instead, we selected the water points purposively [30]. Therefore the water samples tested were not representative of all water points in the area. We did not test the water samples for *V. cholerae* due to logistical and laboratory-capacity constraints. Also, we did not demonstrate a geographical association between the cases and the area served by the GWFS pipeline because we were unable to obtain the coordinates of the pipeline system. Such data would have strengthened our findings.

## Conclusion

Our investigation revealed that cross-border trading might have introduced cholera into the community; subsequently, drinking unsafe water (i.e., water that was not treated or boiled) contaminated by sewage might have facilitated the transmission of the disease, causing a prolonged, community-wide cholera outbreak. We recommended to the public health authorities to disinfect and properly dispose of patients’ faeces, fix the leaking pipeline, chlorinate the piped water system, and conduct health education on boiling and chlorinating drinking water. The outbreak stopped within 6 weeks after these recommendations were implemented.

## Abbreviations

AR: Attack rate
CI: Confidence Interval
DRC: Democratic Republic of Congo
GWFS: Gravitational Water Flow Scheme
IDSR: Integrated Disease Surveillance and Response
MoH: Uganda Ministry of Health
MPN: Most Probable Number
OR_M-H_: Mantel-Haenszel odds ratio
SCD: Standard Case Definition
WASH: Water Sanitation and Hygiene
